# Expected clinical impact of the differences between planned and delivered dose distributions in helical tomotherapy for treating head and neck cancer using helical megavoltage CT images

**DOI:** 10.1120/jacmp.v10i3.2969

**Published:** 2009-07-21

**Authors:** Panayiotis Mavroidis, Sotirios Stathakis, Alonso Gutierrez, Carlos Esquivel, Chenyu Shi, Nikos Papanikolaou

**Affiliations:** ^1^ Department of Medical Radiation Physics Karolinska Institutet and Stockholm University Stockholm Sweden; ^2^ Department of Medical Physics Larissa University Hospital Larissa Greece; ^3^ Department of Radiological Sciences University of Texas Health Sciences Center San Antonio TX USA

**Keywords:** Helical Tomotherapy, conformal radiotherapy, IMRT, biologically effective uniform dose, complication‐free tumor control, dose‐response relations, radiobiological evaluation, MVCT

## Abstract

Helical Tomotherapy (HT) has become increasingly popular over the past few years. However, its clinical efficacy and effectiveness continues to be investigated. Pre‐treatment patient repositioning in highly conformal image‐guided radiation therapy modalities is a prerequisite for reducing setup uncertainties. A MVCT image set has to be acquired to account for daily changes in the patient's internal anatomy and setup position. Furthermore, a comparison should be performed to the kVCT study used for dosimetric planning, by a registration process that results in repositioning the patient according to specific transitional and rotational shifts. Different image registration techniques may lead to different repositioning of the patient and, as a result, to varying delivered doses. This study aims to investigate the expected effect of patient setup correction using the Hi·Art TomoTherapy system by employing radiobiological measures such as the biologically effective uniform dose $\left(\bar{\bar{D}}\right)$ and the complication‐free tumor control probability $\left(P_{+}\right)$. In this study, a typical case of lung cancer with metastatic head and neck disease was investigated by developing a Helical Tomotherapy plan. For the TomoTherapy Hi·Art plan, the dedicated tomotherapy treatment planning station was used. Three dose distributions (planned and delivered with and without patient setup correction) were compared based on radiobiological measures by using the $P_{+}$ index and the $\bar{\bar{D}}$ concept as the common prescription point of the plans, and plotting the tissue response probabilities against the mean target dose for a range of prescription doses. The applied plan evaluation method shows that, in this cancer case, the planned and delivered dose distributions with and without patient setup correction give a $P_{+}$ of 81.6%, 80.9% and 72.2%, for a $\bar{\bar{D}}$ to the planning target volume (PTV) of 78.0Gy, 77.7Gy and 75.4Gy, respectively. The corresponding tumor control probabilities are 86.3%, 85.1% and 75.1%, whereas the total complication probabilities are 4.64%, 4.20% and 2.89%, respectively. HT can encompass the often large PTV required while minimizing the volume of the organs at risk receiving high dose. However, the effectiveness of an HT treatment plan can be considerably deteriorated if an accurate patient setup system is not available. Taking into account the dose‐response relations of the irradiated tumors and normal tissues, a radiobiological treatment plan evaluation can be performed, which may provide a closer association of the delivered treatment with the clinical outcome. In such situations, for effective evaluation and comparison of different treatment plans, traditional dose based evaluation tools can be complemented by the use of $P_{+}-\bar{\bar{D}}$ diagrams.

PACS number: 87.55.Qr Quality assurance in radiotherapy

## I. INTRODUCTION

Dynamic dose delivery using advanced methods for modulating the dose distribution to tumors and organs at risk has recently been developed. Furthermore, accurate imaging is a prerequisite for adaptive radiation therapy of mobile tumors. In order to investigate the effectiveness of such a radiation modality in conjunction with a patient setup correction method, Helical Tomotherapy (HT) was employed using the Helical Megavoltage CT (MVCT) scanner of the tomotherapy unit. Due to the highly conformal distributions that can be obtained with HT, any discrepancy between the intended and delivered dose distributions would likely affect the clinical outcome. Consequently, there is a need to measure those differences in terms of a change in the expected clinical outcome.

The clinical results of radiotherapy strongly depend on the ability of the quality control to identify potential sources of errors, which can be taken into account during planning for treatment. Accuracy in patient positioning is a prerequisite to ensure agreement between the calculated and the delivered dose distribution to the patient but may well be one of the weakest parts of the radiotherapy process.^(^ ^1^ ^–^ ^4^ ^)^ Variations in dose distribution and in dose delivery can contribute to underdosage of the tumor or overdosage of normal tissue, which are potentially related to a reduction of local tumor control and an increase of side effects, respectively. Such variations in delivered dose distribution can be a consequence of patient setup inaccuracies. To identify localization errors in patient setup, portal films or electronic portal imaging devices have long been used for the verification of field alignment.^(5)^ To measure the accuracy of the shifts in patients treated on a helical tomotherapy machine, a megavoltage computed tomography (MVCT) scan has been developed for daily correction of patient positioning.^(^ ^6^ ^–^ ^11^ ^)^

Isodose distribution, dose volume histogram (DVH) and maximum, minimum, mean and standard deviation of a dose distribution, are currently the main concepts for radiotherapy treatment plan evaluation. However, all these evaluation tools are only dose‐based and they do not take the radiobiological characteristics of tumors or normal tissues into account. In some situations, competing plans are characterized by significantly different radiobiological outcomes even though they have similar mean, maximum and minimum doses.^(12)^ To deal with these cases, the use of the biologically effective uniform dose $\left(\bar{\bar{D}}\right)$ and complication‐free tumor control $\left(P_{+}\right)$ have been proposed as alternative plan evaluation tools.^(13)^ The concept of biologically effective uniform dose $\bar{\bar{D}}$ assumes that any two dose distributions are equivalent if they cause the same probability for tumor control or normal tissue complication.^(12)^ The $\bar{\bar{D}}$ concept makes use of the fact that probabilities averaged over both dose distribution and organ radiosensitivity are more relevant to the clinical outcome.

Helical Tomotherapy is a recently developed technology for radiation therapy, which is characterized by treatment plans of higher dose conformality than other IMRT techniques.^(^ ^14^ ^,^ ^15^ ^)^ In HT technology, radiation is delivered helically through fifty‐one projections per rotation. A new module of the TomoTherapy software (TomoTherapy, Inc, Madison, WI), called Planned Adaptive, is employed in this study. With this module, eventual dose discrepancies that may have occurred during treatment can be evaluated and corrected. In this process, the delivered dose can be calculated by using the sinogram for each delivered fraction and the registered MVCT image set (TomoImage) that best corresponds to the patient position for that fraction. The calculation can be done for one or several treatment fractions, which is subsequently compared to the planning dose for the same number of fractions.

The goal of this study is to use radiobiological measures to compare the expected clinical impact of delivering an HT treatment plan with and without accounting for patient setup correction. For this purpose, a representative case of lung cancer with metastatic disease in the head and neck region was selected. For this cancer type, a Helical Tomotherapy plan was developed. Two dose distributions were calculated using the MVCT image sets before and after the patient setup correction. To do that, the fractional dose distributions by the majority (or all) of the fractions were added and renormalized to the total number of fractions planned. To quantify the differences between the planned dose distribution against the delivered dose distributions with and without patient setup correction, the biologically effective uniform dose $\left(\bar{\bar{D}}\right)$ and the complication‐free tumor control probability $\left(P_{+}\right)$ were used, together with more common dosimetric statistical measures, leading to a more complete and comprehensive comparison of the dose distributions.

## II. MATERIALS AND METHODS

### A. Treatment planning and delivery units

The patient was a 75‐year‐old male with extensive‐stage, small‐cell lung cancer and evidence of metastatic disease to the lymph nodes and bone marrow. The head and neck site of the metastatic disease occupied from the right occiput down toward the left neck and left supraclavicular area. Helical Tomotherapy was utilized because of the diffuse fatty involvement and its ability to spare adjacent sensitive organs at risk (OARs). We chose to evaluate a palliative case, which is characterized by an extensive target and organs at risk that are typically located very close to the target. In this way, the clinical problem at hand demands for a dose distribution of very high conformity. It is for this kind of cases that the MVCT method is most useful, since even small misalignments between the patient and the incoming beams can deteriorate significantly the effectiveness of the treatment. Nevertheless, in order to make the analysis more complete, the target was assumed to have different radiosensitivities. Therefore, different strengths of dose were used: one that is used in radical treatments and one that is more appropriate in palliative treatments.

The patient was scanned by a computed tomography (CT) system (LightSpeed, GE Medical Systems, Milwaukee, WI) using axial scan mode. CT scan implemented 1.25 mm slice thickness over the region. Anatomical contours were delineated using the Advantage Simulation software (GE Medical Systems, Milwaukee, WI). A 2 mm volumetric margin expansion was applied to the visible metastases in order to cover microscopic disease and create the PTV which, in this study, is the same as the CTV. CT images and associated contours were transferred to the TomoTherapy TPS (TomoTherapy Inc, Madison, WI).^(16)^

Helical Tomotherapy allows the delivery of image‐guided, intensity‐modulated radiation therapy (IG‐IMRT).^(17)^ Details of the inverse planning algorithm used in tomotherapy have been previously described.^(18)^ Tomotherapy optimization is guided using several parametersthat are exclusively related to Helical Tomotherapy. The user defines the prescription to the tumor structures, and field width is defined by the primary jaws, modulation factor (MF), pitch, and resolution of the calculated dose grid. In tomotherapy, the field width is defined as the slice thickness of the radiation field projected at the isocenter along the gantry rotation axis. Current Hi·ART machines typically have three commissioned field widths of approximately 1.0, 2.5, and 5.0 cm. The modulation factor is defined as the ratio of the maximum leaf opening time to the average opening time of all of the non‐zero leaf opening times. The pitch is defined as the ratio of the distance that the couch travels per rotation to the field width at the gantry axis.

For this study, the plans were generated using a 2.5 cm field width to obtain the most conformal treatment plan deliverable in a reasonable time period. Pitches equivalent to 0.866 divided by an integer were used in this study based on the thread effect work by Kissick et al.^(19)^ For ail plans, the modulation factor was set to 2.0. In general, a higher modulation factor facilitates higher dose gradients. Higher modulation factors lead to longer treatment times. For the examined cancer type, it was assumed that the tumor is more radioresistant than normal in order to force the optimization algorithm to find a very conformal dose distribution that would irradiate the PTV to a high dose. For this reason, the planned and MVCT dose distributions were scaled to a prescription dose and fractionation schedule of 60.0 Gy to 95% volume of the PTV delivered in 30 treatment fractions.

During the planning process, the TomoTherapy TPS downsampled the CT image resolution to 128×128pixels in each slice and increased slice width from 1.25 mm to 2.5 mm over the entire CT image volume set. This was necessary to reduce the amount of memory required for optimization. According to the physics manual of the TomoTherapy TPS and Dose in‐Air Outside the Patient, the following issues should be clarified. Dose is deposited in air when X rays interact with the particles in air. Contrary to some treatment planning systems, which discard this information, the TomoTherapy TPS calculates dose in‐air to improve calculations of skin dose. To save time, dose in‐air is not tabulated and displayed during optimization, except inside the patient (e.g., rectal gas). Nevertheless, attenuation and scatter of rays as they pass through the air is taken into account during optimization, in order to accurately calculate patient dose. Dose in‐air is tabulated, and isodose contours are displayed, at the fractionation stage.

Before each treatment the patient was scanned using the Megavoltage CT (MVCT) capability of Hi‐Art TomoTherapy. The daily MVCT was then fused with the planning CT based on fiducial markers. From the fusion of the two image sets, the daily translations in all three directions were computed and were then applied in order to reposition the patient. From the MVCT images, patient roll can be identified and taken into account during treatment.

For each fraction, the translational data that collected from the fusion process were used along with that fraction sinogram in order to calculate the dose to the patient. Another dose calculation was performed for each fraction using each fraction's sinogram but no patient position correction. The dose distributions for each fraction with and without patient repositioning were computed, and the final DVHs for both dose calculations were compared against the planning ones. Furthermore, to investigate the impact of the presented methodology in radical treatments, three additional clinical cases were examined for verifying the general conclusions of the present analysis. These cases refer to lung, pancreas and prostate cancer patients, who were treated with prescribed doses of 54 Gy, 45 Gy and 74 Gy in 30, 25 and 37 fractions.

### B. Radiobiological treatment plan evaluation

In this study, the dose‐response relations of the tumors and normal tissues are described by the Linear‐Quadratic‐Poisson model. This model can take into account the fractionation effects that are introduced by the irradiation schedule:

(1) $$P(D)=\exp\left(-e^{e\gamma -\left(D/D_{50}\right)(e\gamma -\ln\ln2)}\right)$$

where *P(D*) is the probability to control the tumor or induce a certain injury to a normal tissue that is irradiated uniformly with a dose $D.D_{50}$ is the dose which gives a 50% response and γ is the maximum normalized dose‐response gradient. Parameters $D_{50}$ and γ are specific for every organ and type of clinical endpoint and they can be derived directly from clinical data.^(^ ^20^ ^–^ ^23^ ^)^ When the values of the $D_{50}$ and γ parameters are derived from clinical materials, the dose distributions have to be corrected for the fractionation effects, which means that the knowledge of the α/β ratio is necessary.

The response of a normal tissue to a non‐uniform dose distribution is given by the relative seriality model. This model accounts for the volume effect. For a heterogeneous dose distribution, the overall probability of injury $P_{I}$ (I denotes injury),^(^ ^24^ ^,^ ^25^ ^)^ for a number of OARs is expressed as follows:

(2) $$P_{I}=1-\prod_{j=1}^{N_{organs}}\left(1-P_{I}^{j}\right)=1-\prod_{j=1}^{N_{organs}}\left(1-{\left[1-\prod_{i=1}^{M_{j}}{\left(1-P^{j}{\left(D_{i}\right)}^{s_{j}}\right)}^{\Delta v_{i}}\right]}^{1/s_{j}}\right)$$

where $P_{I}^{j}$ is the probability of injuring organ *j* and $N_{\text{organs}}$ is the total number of vital OARs. $P^{j}(D_{i})$ is the probability of response of the organ *j* having the reference volume and being irradiated with a dose $D_{i}$ as described by Eq. (1). $\Delta v_{i}=\Delta V_{i}/V_{\text{ref}}$ is the fractional subvolume of the organ that is irradiated compared to the reference volume for which the values of $D_{50}$ and γ have been calculated. $M_{j}$ is the total number of voxels or subvolumes in the organ *j*, and $s_{j}$ is the relative seriality parameter that characterizes the internal organization of that organ. s≈1 corresponds to a completely serial structure which becomes non‐functional when at least one functional subunit is damaged, whereas s≈0 corresponds to a parallel structure, which becomes non‐functional when all its functional subunits are damaged. It should be mentioned that other models such as (LKB^(^ ^26^ ^–^ ^28^ ^)^, Parallel^(29)^, etc.) could also have been used with the appropriate response parameter sets.

In tumors, the structural organization is assumed to be parallel since the eradication of all their clonogenic cells is required. Furthermore, in complex multi‐target cancer cases, the eradication of every individual tumor has to be achieved. This pattern indicates that the different targets are related through a parallel organization fashion. Taking these features into account, the overall probability of benefit $P_{B}$ (B denotes benefit from tumor control) is given by the expression:

(3) $$P_{B}=\prod_{j=1}^{N_{tumors}}P_{B}^{j}\prod_{j=1}^{N_{tumors}}\left(\prod_{i=1}^{M_{j}}P^{j}{\left(D_{i}\right)}^{\Delta v_{i}}\right)$$

where $P_{B}^{j}$ is the probability of eradicating tumor *j* and $N_{\text{tumois}}$ is the total number of tumors or targets involved in the clinical case.

In Table 1, the dose‐response parameters of the organs used in this study are shown. This parameter set is based on published data.^(^ ^20^ ^–^ ^23^ ^,^ ^30^ ^–^ ^33^ ^)^ For many tissues, the values of relative seriality have been determined from clinical data in the work by Emami et al,^(20)^ where the relative seriality model was fit on the dose‐response diagrams (see Ågren 1995).^(21)^ The uncertainties that are associated with these parameters define the confidence interval of the entire dose‐response curve around its best estimate.^(34)^ In this study, it was assumed that the patient is of average radiosensitivity, which is characterized by the mean estimates of the radiobiological parameters presented.

**Table I:** Summary of the model parameter values for the head and neck cancer case. $D_{50}$ is the 50% response dose, γ is the maximum normalized value of the dose‐response gradient and s is the relative seriality, which characterizes the volume dependence of the organ.

*Relative Seriality Model*	*Parameter Values*			
*Head & Neck Cancer*	$D_{50}(Gy)$	γ	*s*	α/β
${\text{PTV}}_{\text{radical}}$	74.1	2.7	—	10.0
${\text{PTV}}_{\text{palliative}}$	52.8	2.3	—	10.0
Spinal Cord	68.6	1.9	4.0	3.0
Left Parotid	26.0	1.8	1.0	3.0
Right Parotid	26.0	1.8	1.0	3.0

The effectiveness of the different treatment plans is evaluated though the concepts of $\bar{\bar{D}}$ and $P_{+}.\bar{\bar{D}}$ is the biologically effective uniform dose,^(^ ^12^ ^,^ ^35^ ^)^ whereas $P_{+}$ is a scalar quantity, which expresses the probability of achieving tumor control without causing severe damage to normal tissues.^(13)^ In current clinical practice, the quality of a treatment plan is usually assessed through the mean doses and dose variations in the target volumes and organs at risk, together with the respective minimum and maximum doses. In addition to these parameters, the $\bar{\bar{D}}$ concept is used in this study to evaluate the treatment plans. The biologically effective uniform dose is defined as the dose that causes the same tumor control or normal tissue complication probability as the actual dose distribution given to the patient. In the former case, the biologically effective uniform dose is denoted as ${\bar{\bar{D}}}_{B}$. The notations $\bar{\bar{D}}$ indicates that the quantity has been averaged over both the dosimetric (dose distribution) and the biological (dose‐response relations) information of the clinical case. The general expression of $\bar{\bar{D}}$ is defined, for a given tumor or normal tissue, from its dose‐response relation without dependence on the radiobiological model used. It is derived numerically by the first part of the following equation, whereas for a tissue of uniform radiosensitivity, $\bar{\bar{D}}$ is given from the latter analytical formula:

(4) $$P(\vec{D})\equiv P(\bar{\bar{D}})\Rightarrow \bar{\bar{D}}=\frac{e\gamma -\ln(-\ln(P(\vec{D})))}{e\gamma -\ln(\ln2)}$$

where $\bar{\bar{D}}$ denotes the 3‐dimensional dose distribution. This definition is a generalization and combination of the effective uniform dose $D_{\text{eff}}$ introduced by Brahme,^(36)^ and the equivalent uniform dose (EUD) introduced by Niemierko.^(37)^ Using this dose concept, a number of plan trials can be compared based on radiobiological endpoints by normalizing their dose distributions to a common prescription point $\left(\bar{\bar{D}}\right)$.

In this work, the probability of getting benefit from a treatment (tumor control) is denoted by $P_{B}$, whereas the probability for causing severe injury to normal tissues by $P_{I}$,^(^ ^25^ ^,^ ^35^ ^)^ Using these quantities, $P_{+}$ can be estimated from the following expression:

(5) $$P_{+}=P_{B}-P_{B\cap I}=P_{B}-P_{I}+\delta (1-P_{B})P_{I}$$

where the parameter δ represents the fraction of the patients with statistically independent tumor and normal tissue responses. A set of clinical data estimated the value of δ being below 20%.^(21)^ The importance of this concept increases with the accuracy of the radiobiological parameters, which describe the dose‐response relations of the different tumors and normal tissues since this makes the probabilities $P_{B}$ and $P_{I}$ more reliable and clinically relevant. The objective of the radiobiological evaluation was to estimate the normal tissue tolerance against the optimum target dose needed.^(^ ^38^ ^,^ ^39^ ^)^ An in‐house developed software tool was used to calculate the dose volume histograms (DVHs) and the probabilities of benefit and injury, as well as the values of the complication‐free tumor control probability, $P_{+}$ and biologically effective uniform dose, $\bar{\bar{D}}$.

## III. RESULTS

**Figure 1:**
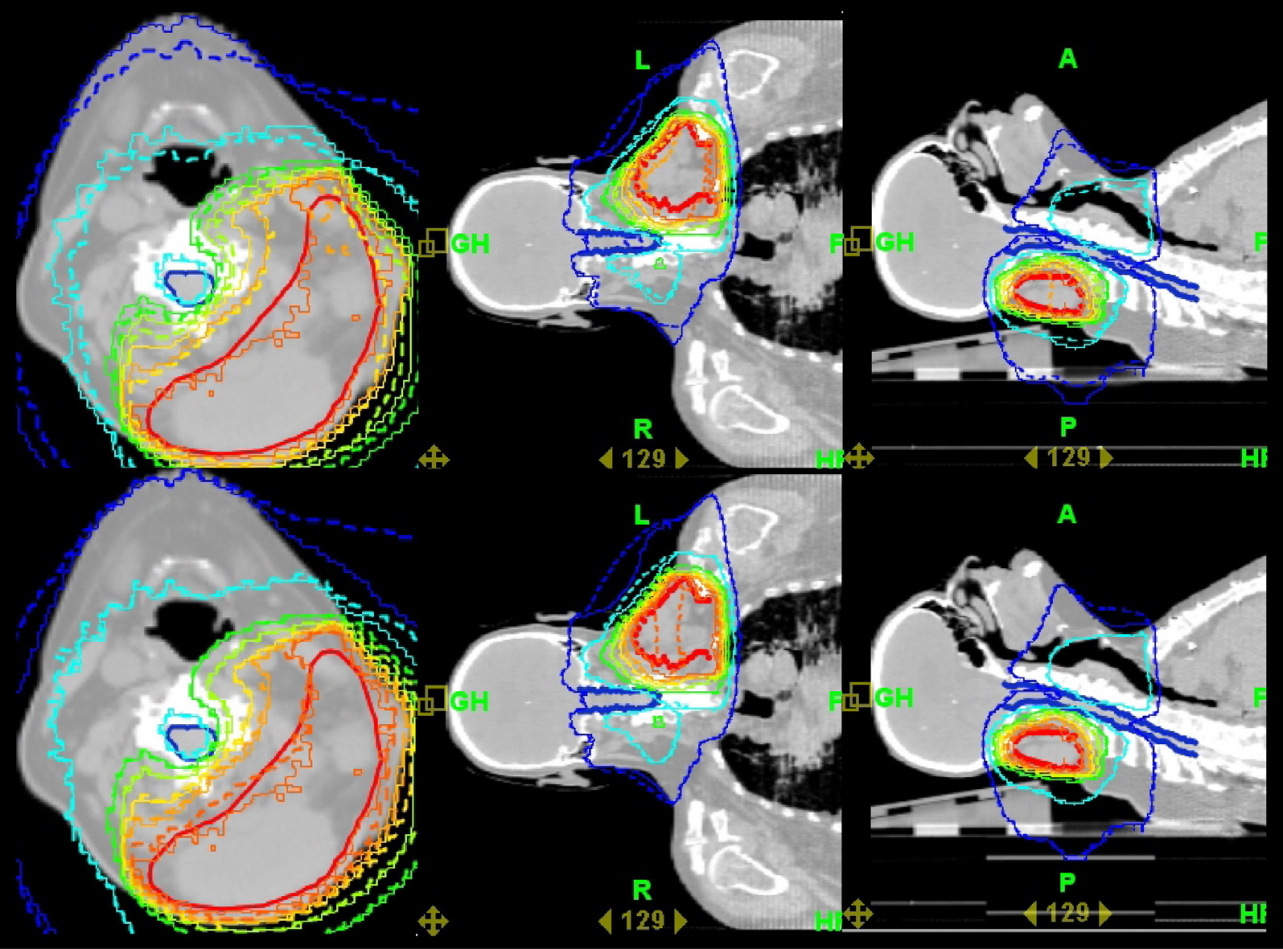
The reference CT slice of a head and neck cancer patient is shown for the planned and delivered dose distributions of the Helical Tomotherapy treatment plan in transverse coronal and sagittal planes. The anatomical structures involved are illustrated together with the applied dose distributions to be delivered to the patient. Upper panel: the isodose distributions of the plan (solid) and the MVCT dose distribution without patient setup correction (dashed); lower panel: the isodose distributions of the plan (solid) and the MVCT dose distribution with patient setup correction (dashed).

Figure 1 depicts the comparison of the isodose curve distributions of the HT plan against the MVCT dose distributions (with and without patient setup correction), in transverse, coronal and sagittal views. In this clinical case, the sensitive organs at risk are the spinal cord, the left and right parotids. Based on the isodose curve distributions, it is clear that the dose distribution without setup correction has a quite larger dose spread outside the PTV and a larger inhomogeneity inside the PTV, compared to the planned dose distribution. The mean dose to the PTV was highest in the HT plan and lowest in the dose distribution without setup correction (Table 2.

**Table 2:** Summary of the dosimetric comparison for the three dose distributions of the head and neck cancer case.

TISSUE	${\bar{\bar{D}}}_{p}$	${\bar{\bar{D}}}_{\text{Width}}$	${\bar{\bar{D}}}_{W/o}$	${\bar{D}}_{p}$	${\bar{D}}_{\text{Width}}$	${\bar{D}}_{W/o}$	$D_{P}^{\max}$	$D_{\text{Width}}^{\max}$	$D_{W/o}^{\max}$	$D_{p}^{\min}$	$D_{\text{Width}}^{\min}$	$D_{W/o}^{\min}$
PTV	60.6	60.4	55.1	60.6	60.5	56.4	62.0	64.7	65.4	58.4	57.0	49.8
Right Parotid	11.2	11.4	12.1	8.7	8.8	9.1	11.8	12.0	13.4	6.4	7.0	6.6
Left Parotid	15.0	15.1	16.1	9.3	9.9	10.0	18.2	17.0	19.4	6.1	6.2	6.5
Spinal Cord	43.7	44.7	41.5	16.8	17.2	17.2	46.1	48.4	42.9	3.1	2.3	3.0

Note: The indices *P*, *With* and *W/o* refer to ‘Planned’, ‘With setup correction’, and ‘W/o setup correction’, respectively.

More specifically, the dose distribution from the plan and those from the MVCT with and without setup correction deliver to the PTV 60.6 Gy, 60.5 Gy and 56.4 Gy, respectively. The respective dose variations in the PTV are 58.4–62.0 Gy, 57.0–64.7 Gy and 49.8–65.4 Gy showing that, in the former case, the dose distribution is more homogeneous. Regarding the organs at risk, the MVCT dose distributions deliver higher mean doses compared to the HT plan. More specifically, the dose distribution from the plan and those from the MVCT with and without setup correction deliver 8.7 Gy, 8.8 Gy and 9.1 Gy to the right parotid, 9.3 Gy, 9.9 Gy and 10.0 Gy to the left parotid, and 16.8 Gy, 17.2 Gy and 17.2 Gy to the spinal cord, respectively. It is shown that in all cases the mean dose $\bar{D}$ to the PTV is very close to the corresponding $\bar{\bar{D}}$ values because of the relatively small dose variations within the PTV. On the contrary, the dose distributions within the OARs are characterized by much larger variations. This has a consequence that the $\bar{\bar{D}}$ values of the normal tissues are significantly greater than the corresponding mean doses $\bar{D}$, especially for the left parotid and spinal cord.

**Figure 2:**
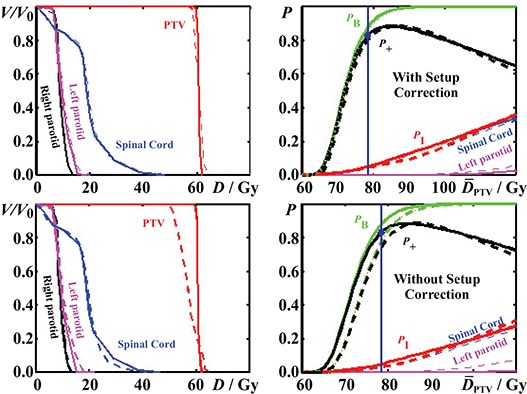
Left: the DVHs of the PTV and those of the organs at risk (spinal cord, left and right parotids); Right: the prescription dose levels. The dose‐response curves that are derived from the radiobiological evaluation of the three dose distributions are plotted using the mean dose to the PTV on the dose axis.

In Fig. 2, the treatment plans are compared in terms of DVHs and dose‐response curves. The dose‐response curves of the PTV and each organ at risk are presented, together with the $P_{+}$ curve. In the right diagrams of Fig. 2, the response curves are all normalized to the mean dose in the $\text{PTV}\;\left({\bar{D}}_{\text{ITV}}\right)$. In these diagrams, the curves show how tissue responses change with dose prescription since the same dose distribution is kept at all dose levels. This approach gives emphasis to the therapeutic window that characterizes each treatment plan. A quantitative summary of the physical and biological comparisons is presented in Tables 2 and 3.

**Table 3:** Summary of the radiobiological comparison for the head and neck cancer case. The three dose distributions examined are denoted as ‘Planned’, ‘With setup correction’ and ‘W/o setup correction’. The results refer to a 60 Gy dose prescription of a palliative PTV and a dose prescription producing less than 5% complication rate in a radioresistant PTV treatment.

*Dose Distribution*	*Planned*		*With setup correction*		*W/o setup correction*	
*Dose Prescription*	*60 Gy*	$P_{I}<5\%$	*60 Gy*	$P_{I}<\;5\%$	*60 Gy*	$P_{I}<\;5\%$
*TISSUE*	*P* (*%*)		*P* (*%*)		*P* (*%*)	
PTV	75.4	86.3	75.1	85.1	72.3	75.1
Right Parotid	0.0	<0.01	0.0	<0.01	0.0	<0.01
Left Parotid	0.0	<0.01	<0.01	0.01	<0.01	0.15
Spinal Cord	0.04	4.6	0.08	4.2	0.02	2.7
$P_{+}\;(\%)$	75.4	81.6	75.0	80.9	72.2	72.2
${\bar{D}}_{\text{PTV}}\;(\text{Gy})$	60.0	78.0	60.0	78.0	60.0	78.0
$P_{B}\;(\%)$	75.5	86.3	75.1	85.1	72.3	75.1
$P_{I}\;(\%)$	0.04	4.64	0.08	4.20	0.02	2.89
${\bar{\bar{D}}}_{B}(\text{Gy})$	60.0	78.0	59.9	77.7	59.1	75.4

For the planned dose distribution and at the dose prescription where the expected complication rates are below 5%, the value of $P_{+}$ was 81.6%, for a mean dose to the PTV of $\bar{D}=78.0\text{Gy}$ and biologically effective uniform dose, ${\bar{\bar{D}}}_{\text{PTV}}$ of 78.0 Gy. The total control probability $P_{B}$ was 86.3% and the total risk for complications $P_{I}$ was 4.64%. For the delivered dose distribution with patient setup correction, the value of $P_{+}$ was 80.9% for $\bar{D}=78.0\text{Gy}$ and ${\bar{\bar{D}}}_{\text{PTV}}$ of 77.7 Gy. The response probabilities $P_{B}$ and $P_{I}$ were 85.1% and 4.20%, respectively. Finally, for the delivered dose distribution without patient setup correction in every fraction using the MVCT method, the value of $P_{+}$ was 72.2% for $\bar{D}=78.0\text{Gy}$ and ${\bar{\bar{D}}}_{\text{PTV}}$ of 75.4 Gy. The response probabilities $P_{B}$ and $P_{I}$ were 75.1% and 2.89%, respectively. According to these results, the expected clinical effectiveness of the treatment plan dropped by a $\Delta P_{+}$ of 9.4% in the case where no patient setup correction is performed, and 0.7% if patient alignment is corrected in every fraction of the treatment.

Using the radiobiogical parameters of a less radioresistant target as that involved in a palliative treatment, then the results of the radiobiological analysis at the prescription level of 60 Gy would be as follows: For the planned dose distribution, the value of $P_{+}$ was 75.4%, ${\bar{\bar{D}}}_{\text{PTV}}=60.0\;\text{Gy},\;P_{B}=75.5\%$ and $P_{I}=0.04\%$. For the delivered dose distribution with patient setup correction, the value of $P_{+}$ was 75.0%, ${\bar{\bar{D}}}_{\text{PTV}}=59.9\;\text{Gy},\;P_{B}=75.1\%$ and $P_{I}=0.08\%$. Finally, for the delivered dose distribution without patient setup correction, the value of $P_{+}$ was 72.2%, ${\bar{\bar{D}}}_{\text{PTV}}=59.1\;\text{Gy},\;P_{B}=72.3\%$ and $P_{I}=0.02\%$.

The reason for observing a lower TCP as well as a lower spinal cord complication rate is the shift of the applied dose distribution away from the spinal cord. As a consequence, parts of the target are underdosed compared to the initially prescribed criteria, whereas the spinal cord receives lower dose at the expense of the dose received by the left parotid. The fact that the MVCT method corrected the delivered dose distribution back to the planned one shows the good properties of the method. On the other hand, this is an example of the importance of using radiobiological measures in these processes because they can provide means of interpreting differences observed in DVH charts in clinical terms.

In the three radical cases, which are characterized by smaller PTVs, the differences between the planned and patient setup corrected dose distributions are very small, leading to almost identical radiobiological results. For the lung cancer case, at the optimum dose levels of the dose distributions, with and without patient setup correction, the complication‐free tumor control probabilities $P_{+}$ are 56.8% and 57.6% for a ${\bar{\bar{D}}}_{\text{PTV}}$ of 65.0 Gy. The respective total control probabilities, $P_{B}$ are 78.1% and 78.1%, whereas the corresponding total complication probabilities, $P_{I}$ are 21.3% and 20.5%. For the pancreas cancer case, at the optimum dose levels of the two dose distributions, the $P_{+}$ values are 97.5% and 94.6% for a ${\bar{\bar{D}}}_{\text{PTV}}$ of 70.0 Gy, respectively. The respective $P_{B}$ values are 98.7% and 98.7%, whereas the corresponding $P_{I}$ values are 1.2% and 4.1%. For the prostate cancer case, at the optimum dose levels of the two dose distributions, the $P_{+}$ values are 55.9% and 57.7% for a ${\bar{\bar{D}}}_{\text{PTV}}$ of 90.0 Gy, respectively. The respective $P_{B}$ values are 84.7% and 83.7%, whereas the corresponding $P_{I}$ values are 28.8% and 26.1%.

## IV. DISCUSSION

Quality control is of outmost importance in radiation therapy because of the existence of many potential sources of errors. Such errors, which take place during the delivery of the treatment to the patient, have as a result the degradation of the curative power and effectiveness of the treatment.^(40)^ Positioning uncertainties and breathing effects are such sources of errors because they lead to a dose delivery that is different than the one intended originally to be given.^(^ ^38^ ^,^ ^41^ ^)^

In this study, an extensive‐stage small cell lung cancer with head and neck metastatic disease was investigated in order to evaluate the clinical effectiveness of using megavoltage CT images for daily patient setup. Criteria such as the mean and minimum target dose, normal tissue tolerance doses, isodose levels and DVHs were mostly used in the physical analysis of the three treatment plans.^(42)^ More specifically, the uniformity of dose in the target volume and the dose level constraints were the basis for the evaluation and classification of the different dose distributions. The PTV should receive at least a certain minimum dose specified by the clinical protocols, while the tolerance doses of the involved organs at risk should not be reached. In the HT plan, the PTV is covered sufficiently well by the dose distribution. At the same time, all the OARs are spared very well.

In the comparisons between the different dose distributions, the differences observed on the DVHs are not always reflected in the radiobiological evaluation. This is because different dose‐response relations expressing the radiobiological characteristics of different tissues are affected in different ways by a certain dose distribution. Similarly, two dose distributions may have the same mean dose and standard deviation but different response probabilities when irradiating the same tissue. In the right diagrams of Fig. 2, the classification and superiority of the plans are determined by comparing the response curves of the PTV and organs at risk. By using these kinds of diagrams, it is observed how the control probability changes when certain complication thresholds are examined. This evaluation procedure could be used as a guide for treatment plan evaluation and dose prescription. The biological evaluation plot of a dose plan is a good illustration of the expected clinical outcome, as the dose volume histogram chart is a good illustration of the volumetric dose distribution delivered to the patient. Using the dose‐response diagrams together with the dosimetric diagrams, a more complete picture of the delivered treatment is given.

As it is shown in Fig. 2, the expected complication‐free tumor control for the planned dose distribution is better than the delivered dose distributions with and without setup correction over the range of clinical prescribed doses. The reason for this is that the HT plan irradiates the PTV a little more effectively, compared to the delivered dose distribution with setup correction and much more effectively than the delivered dose distribution without setup correction. On the other hand, spinal cord is spared similarly by the first two dose distributions, whereas it is spared even better by the dose distribution without setup correction, as is indicated by the response probabilities of spinal cord and the corresponding $\bar{\bar{D}}$ values (Table 2. In comparison with the dose distribution without setup correction, both the planned and delivered with setup correction dose distributions show significantly higher target control and slightly higher complication probabilities. It should be mentioned that although the HT plan delivers lower mean dose to the spinal cord compared to the dose distribution without setup correction, it shows a higher complication probability because of its greater maximum dose and the high relative seriality value of spinal cord.

In the right diagrams of Fig. 2 and in Table 3, it is observed that the planned dose prescription is lower than the optimum. This is because at the planned (prescribed) level of the dose distribution, an acceptable compromise has been achieved. However, when the radiobiological data of the different tissues are available, then it can be seen that there is a margin of improvement. By a small increase in the dose prescription, the gain in tumor control is larger than the increase in normal tissue complications, until a balance is reached.

The width of the $P_{+}$ curve expresses the separation between the response curves of the tumors and the involved normal tissues.^(43)^ The width of the therapeutic window also gives an indication of how robust the treatment plan is against dose delivery and patient radiosensitivity uncertainties. The clinical evaluation of a certain dose distribution and the optimization of its dose level can be performed by the use of the complication‐free tumor control index. At the same time, since the most conformal dose distribution generates a higher value of $P_{+}$, the optimum dose delivery is indicated. Instead of only evaluating physical functions, this method is a rather good way for making use of the patient specific characteristics.

It has been shown that radiation doses to OARs can be significantly limited when using Helical Tomotherapy. However, when CTV is adjacent to highly radiosensitive critical organs, the PTV expansion often does not allow for much organ sparing. Margin expansion of the GTV and CTV, as well as dose escalation possibilities, are allowed by preferential OAR sparing. Dose inhomogeneity within an organ is generally associated with the delivery of different daily radiation doses to different regions. The effect of this inhomogeneity is to produce a concurrent radiation local boost that will give a higher total radiation dose to a limited region.^(44)^ A very accurate setup can be provided by taking a CT image immediately before each treatment fraction.^(45)^ In certain cancer types, during treatment delivery, organs such as liver, pancreas and kidneys may move in and out of the PTV due to patient setup uncertainties. The need for quite large setup margins may limit the opportunity to achieve the goals of dose escalation and target volume expansion. Furthermore, in case of a tumor that is shrinking during treatment, an adaptive radiotherapy optimization should be employed where treatment configuration is re‐optimized before each fraction taking into account the dose delivered in all the previous fractions of the treatment. If the tumor is smaller and further away from critical structures, the general conclusions remain, but absolute values of the differences would be much smaller.

**Figure 3:**
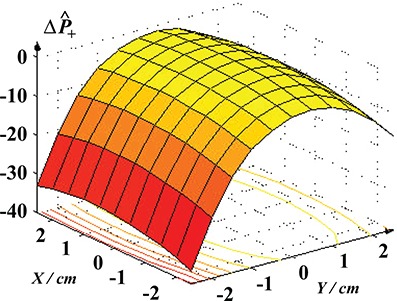
Demonstration of the effects of patient setup on the expected clinical outcome. It is observed that a setup misalignment in the direction where the sensitive organs at risk lie reduces $P_{+}$ rapidly, indicating that conformal plans are very sensitive to positioning errors. If a reliable setup procedure is not available, a less conformal treatment technique could be more effective and secure.

Figure 3 illustrates a study of how the effectiveness of a dose distribution drops when the patient is not aligned correctly with the beam. The study was carried out for a very conformal plan. This phenomenon is more pronounced for the more conformal treatment plans where the setup of the patient is more critical. In this diagram, the dose distribution has been shifted within an 8 cm range in the anterior‐posterior and the sinister‐dexter directions. The values of $P_{+}$ drop much more dramatically if the patient is shifted in the direction where the organs at risk have their proximal borders in direct contact with the PTV.

The results and conclusions of this study are strongly dependent on the accuracy of the radiobiological models and the parameters describing the dose‐response relation of the different tumors and normal tissues. It has to be stated that the values of these dose‐response parameters are normally determined from well‐organized clinical trials, and they express the radiobiological characteristics of a certain type of tumor or organ regarding the manifestation of a certain endpoint. So, if one assumes that the examined tumor is of different stage or the examined normal tissue endpoint is different, then another set of dose‐response parameters has to be used, leading to different results. In our case, by changing the parameters characterizing the radiosensitivity of the target, we realized that the absolute values of the initial observations changed but not the pattern of the observations. Furthermore, it is known that all the existing models are based on certain assumptions or take into account certain biological mechanisms. The determination of the model parameters expressing the effective radiosensitivity of the tissues is subject to uncertainties imposed by the inaccuracies in the patient setup during radiotherapy, lack of knowledge of the inter‐patient and intra‐patient radiosensitivity, and inconsistencies in treatment methodology.^(^ ^46^ ^–^ ^48^ ^)^ Consequently, the determined model parameters (such as the $D_{50}$, γ and *s*) and the corresponding dose‐response curves are characterized by confidence intervals. In the present study, most of the tissue response parameters have been taken from recently published clinical studies, where these parameter confidence intervals has been reduced significantly (e.g., uncertainty of around 5% in the determination of $D_{50}$). In the right diagrams of Fig. 2, the confidence intervals of the $P_{+},\;P_{B}$ and $P_{I}$ response curves are denoted by bars at different response levels (50% and 80% for the PTV, 10% and 25% for normal tissues). It can be observed that the confidence intervals of the different dose distributions overlap, which means that the estimated response differences are smaller than the associated uncertainties. However, this factor does not affect their relative scoring / classification but their association with the treatment outcome that will be clinically registered.

Given that one case of a single treatment site was evaluated in this study, a statistically significant sample of cases would give a more accurate picture of the comparisons. By examining three additional patients who were receiving radical radiotherapy for lung, pancreas and prostate cancers, respectively, it was found that the observations of the present analysis are repeated. More specifically, at the optimum dose levels of the dose distributions with (planned) and without patient setup correction, their difference in the complication‐free tumor control probability $\Delta P_{+}$ ranged from 0.8 to 2.9%, in the tumor control probability $\Delta P_{B}$ ranged from 0.0 to 1.0%, and in the normal tissue complication probability $\Delta P_{I}$ ranged from 0.8 to 2.9%.

As is noted above, the expected complication‐free tumor control for the dose distributions from the plan or with setup correction is not always better than the delivered dose distributions without setup correction. The reason is that the HT TPS does not have the possibility of performing radiobiological treatment plan optimization and the examined treatment plans have not been radiobiologically optimized. Hence, the planned dose distributions did not produce the maximum expected complication‐free tumor control. In all the cases, the PTV is irradiated similarly or a little more effectively by the planned and delivered dose distribution with setup correction compared to the delivered dose distribution without setup correction. This is supported by the tumor control probabilities $P_{B}$ that are presented in Table 4. On the other hand, the setup uncertainties produce higher normal tissue complications when the OARs move into the high dose region (pancreas cancer case) or lower expected responses when the OARs move away from the high dose region (prostate cancer case). (See Table 4)

**Table 4:** Summary of the radiobiological comparison for the lung, pancreas and prostate cancer cases, who receive radical radiotherapy.

*Dose Distribution*	*Planned / With setup correction*	*W/o setup correction*
	*Lung Cancer Case*	
$P_{+}(\%)$	56.8	57.6
${\bar{D}}_{\text{PTV}}(\text{Gy})$	65.0	65.0
$P_{B}\;(\%)$	78.1	78.1
$P_{I}\;(\%)$	21.3	20.5
${\bar{\bar{D}}}_{B}(\text{Gy})$	64.8	64.8
	*Pancreas Cancer Case*	
$P_{+}(\%)$	97.5	94.6
${\bar{D}}_{\text{PTV}}(\text{Gy})$	70.0	70.0
$P_{B}\;(\%)$	98.7	98.7
$P_{I}\;(\%)$	1.2	4.1
${\bar{\bar{D}}}_{B}(\text{Gy})$	69.9	69.9
	*Prostate Cancer Case*	
$P_{+}(\%)$	55.9	57.7
${\bar{D}}_{\text{PTV}}(\text{Gy})$	90.0	90.0
$P_{B}\;(\%)$	84.7	83.7
$P_{I}\;(\%)$	28.8	26.1
${\bar{\bar{D}}}_{B}(\text{Gy})$	90.6	90.2

According to these results, the expected clinical effectiveness of the planned and delivered dose distributions with versus without patient setup correction can change by a maximum $\Delta P_{+}$ of around 3% in medium sized tumors and around 9% in large sized tumors. However, this was expected, since the different treatment plans were produced using optimization algorithms which calculated the final dose distributions based on predefined and excessively demanding criteria (inverse optimization), while the delivered dose distributions were based on a large number of patient setup registrations. In this way, it was ensured that a statistically significant sample of cases would give a very similar picture of the comparisons. The potentially superior radiobiological results of the planned and delivered with setup correction dose distributions against the dose distribution without setup correction can be even better demonstrated by using more severe endpoints in normal tissue complications. Furthermore, the findings of this paper show that the $P_{+}$ and $\bar{\bar{D}}$ concepts are very useful in comparing conformal dose distributions, and they can give useful information regarding the clinical impact of the discrepancies in dose delivery.

## V. CONCLUSIONS

In this study, the clinical effectiveness of planned and delivered dose distributions was evaluated for a treatment in the head and neck region. Using both physical and biological criteria, this evaluation showed that the dose distribution without patient setup correction has a poorer expected clinical outcome as compared to the HT plan and the delivered dose distribution with setup correction. The difference between the latter two dose distributions was small. The therapeutic indices $P_{+}$ and $\bar{\bar{D}}$ were used as figures of merit for the different dose distributions and it was shown that their use may increase the likelihood of accomplishing a good treatment result. A closer association of the delivered treatment with the clinical outcome and additional information about the fitness of the dose distribution with the radiosensitivity map of the patient is provided by the radiobiological treatment plan evaluation. Such an evaluation takes into account the dose‐response characteristics of the irradiated targets and normal tissues involved in a given clinical case. The concept of $\bar{\bar{D}}$ was used to provide a proper dose prescription basis for comparing treatment plans through evaluation of the biological effects of the dose distributions. The application of the $P_{+}$ and $\bar{\bar{D}}$ concepts on the examined dose distributions revealed differences in their expected therapeutic impact. Based on this study, it can be concluded that clinical cases, which may look dosimetrically similar, can be quite different in radiobiological terms. The more conformal a treatment technique, the more precise and accurate the patient setup process should be. In these techniques, the dose distribution is so well matched with the radiosensitivity map of the clinical case that a small misalignment in the setup can greatly reduce the effectiveness of the therapy. If a reliable positioning procedure is not available, a less conformal technique could be more effective and trustworthy. The quality of a treatment does not only depend on the conformity of the applied technique but also on the quality of the supporting services.
